# The impact of chronic airway disease on symptom severity and global suffering in Canadian rhinosinusitis patients

**DOI:** 10.1186/s40463-018-0287-6

**Published:** 2018-05-29

**Authors:** Kimberly Luu, Jason Sutherland, Trafford Crump, Giuping Liu, Arif Janjua

**Affiliations:** 1 Division of Otolaryngology – Head & Neck Surgery, University of British Columbia, Gordon and Leslie Diamond Health Care Centre, DHCC, Vancouver General Hospital, 4th. Floor Rm. 4299B 2775 Laurel Street, Vancouver, BC V5Z 1M9 Canada; 2 UBC Centre for Health Services and Policy Research, Vancouver Campus, 201-2206 East Mall, Vancouver, BC V6T 1Z3 Canada; 3 University of Calgary, 2500 University Dr. NW, Calgary, AB T2N 1N4 Canada

**Keywords:** Endoscopic sinus surgery, Comorbidities, Quality of life, Unified airway

## Abstract

**Background:**

Patients with Chronic Rhinosinusitis (CRS) can suffer from a significant decline in their quality of life. CRS patients have a high prevalence of comorbid conditions and it is important to understand the impact of these conditions on their CRS-related quality of life. This study measures the impacts of chronic pulmonary comorbidities on quality of life, pain, and depression scores among patients with CRS awaiting Endoscopic Sinus Surgery (ESS).

**Methods:**

This study is based on cross-sectional analysis of prospectively collected patient-reported outcome data collected pre-operatively from patients waiting for ESS. Surveys were administered to patients to assess sino-nasal morbidity (SNOT-22), depression and pain. The impact of pulmonary comorbidity on SNOT-22 scores, pain and depression was measured.

**Results:**

Two hundred fifthy-three patients were included in the study, 91 with chronic pulmonary comorbidity. The mean SNOT-22 scores were significantly higher among patients with chronic pulmonary comorbidities than among patients without (37 and 48, respectively). This difference is large enough to be clinically significant. Patients with chronic pulmonary comorbidities reported slightly higher depression scores than those without.

**Conclusions:**

This study found that among CRS patients waiting for ESS, chronic pulmonary comorbidities are strongly associated with significantly higher symptom burden.

## Background

At least 5 % of Canadians suffer from Chronic Rhinosinusitis (CRS) [1, 2]. Patients with CRS may experience significant facial pain, nasal congestion, nasal discharge and, or a reduction in their sense of smell [3]. Relative to other chronic diseases, it is estimated that the quality-of-life burden of patients with CRS is comparable to diseases such as congestive heart failure, chronic obstructive pulmonary disease (COPD), angina, and back pain [4]. This decrease in quality of life is the reason patients seek treatment and the goal of treatment is to decrease this symptom burden. In order to better triage and treat CRS patients, we need to identify and explore what factors negatively affect these patients’ quality of life.

Patients with CRS are known to have a significantly higher prevalence of comorbid conditions [5], most commonly asthma and chronic pulmonary diseases [1]. Additionally, the concept of a united airway theory connects disorders of the lower and upper respiratory tract, prompting clinicians to consider the impact of respiratory pathology when managing CRS [6]. It is therefore likely that lower airway diseases will have an effect on the symptomatology of CRS patients. It is important to elaborate on the effect that lower respiratory conditions have on CRS patients, so that identification of these comorbidities will prompt clinicians to treat these patients appropriately and thoroughly.

There is evidence that correlates lower airway disease severity with radiological CRS severity [7]. However, radiologic evidence does not correlate well with clinical symptoms, thus cannot necessarily be used as an indication of symptom burden. Clinical management of CRS is based on disease symptoms and not physical or radiologic findings. Presently, although generally assumed, we do not have much evidence that lower airway disease impacts quality of life, pain, sinonasal symptoms, or depression in CRS patients. Thus, the primary objective of this study is to measure the relationship between chronic pulmonary comorbidities and patients’ sino-nasal symptom severity, pain and depression prior to Endoscopic Sinus Surgery (ESS).

Research on CRS is underrepresented in the literature when taking into account the clinical burden of disease compared to other chronic illnesses such as asthma or diabetes [8]. Dissemination of this information will provide a better understanding of this relationship and thus a more holistic understanding of CRS patients. This information will help inform all aspects of patient management, including: involvement of multidisciplinary teams, identification and management of specific symptoms, and triage decisions about patients who may most benefit from ESS.

## Methods

This study is a prospective cross sectional analysis of patient reported outcomes. Consecutive new patients diagnosed with chronic rhinosinusitis by two tertiary care rhinologists in the Vancouver Coastal Health Authority were prospectively identified. To target patients with quality of life impact from CRS, the patients who failed medical management and consented for endoscopic sinus surgery were recruited for the study. Data collection was initiated in September 2012 and ended April 2016. This study is approved by the University of British Columbia’s Behavioural Research Ethics Board.

Surveys were administered to the enrolled patients through mail or online methods, depending on the preferences of the patient. Response was encouraged through a maximum of three follow-up telephone calls. The survey package included questions regarding patients’ demographic characteristics, such as age and gender. Additional demographic data, including comorbidities, medications, and past surgical history were additionally obtained from the patients through surgical intake forms. Patient reported outcomes data was obtained via instruments for CRS-specific symptoms, depression, and pain. All instruments utilized are validated and widely used in measuring and reporting health-related quality of life outcomes.

### Instruments: SNOT-22, PHQ-9, PEG-3

The Sino-Nasal Outcome Test-22 (SNOT-22) was the instrument used to measure CRS-specific symptoms. The SNOT-22 is a common and well-validated instrument [9, 10], that includes twenty-two items associated with sino-nasal health. Each item is ranked using a Likert scale ranging from 1 (no problem) to 5 (problem as bad as it can be). The rankings are aggregated into a global score that ranges from 22 (best health) to 110 (worst health). A nine-point change in the SNOT-22 global score has demonstrated to be clinically meaningful [9].

Depression was measured using the Patient-Health Questionnaire-9 (PHQ-9) [11]. The PHQ-9 uses nine items to measure domains of depressive symptoms and functional impairment. Each items is ranked from zero (not at all bothered) to three (bothered nearly every day). The rankings are aggregated into a global score that ranges from zero to 27. Higher PHQ-9 scores are associated with more severe depression symptoms, and scores above nine are associated with clinically significant depression [12].

Pain was measured using the PEG-3 instrument [13]. This is a three-item instrument measuring domains of pain intensity and interference. Each item in the PEG-3 is ranked from zero (no pain) to 10 (highest level of pain). The overall score is determined by averaging the items’ rankings, thus ranging from zero to 10. An overall score above three is associated with clinically significant pain [14].

### Analysis

Using the chart review comorbidity data, patients were categorized into one of two groups. The first group was defined as having a chronic pulmonary comorbidity if there was documentation of the following comorbidities: asthma, emphysema, or chronic bronchitis. The second group did not have documentation of any chronic pulmonary comorbidity.

Differences between the two groups in terms of their demographic characteristics were evaluated using Pearson’s chi-square tests. Differences between the two groups in terms of their overall SNOT-22, PHQ-9, and PEG-3 scores were tested using t-tests.

The research question being answered was whether chronic pulmonary comorbidity was associated with poorer health-related quality-of-life. To do this, three multivariate linear regression models were developed to measure the association between a chronic pulmonary comorbidity and the SNOT-22, PHQ-9, and PEG-3 scores, respectively. In the linear models, the effects of age group (defined in 10-year increments), gender, and the number of other comorbid conditions (defined as 0, 1, or ≥ 2, comorbidities other than chronic pulmonary comorbidities) were adjusted for.

Tests of significance of individual variables were evaluated and reported at the 5% level. All variables were retained in the regression models, irrespective of their significance.

## Results

Overall, 34% of eligible CRS patients waiting for elective ESS in VCH returned their survey. Among the study’s patients, 36% reported a chronic pulmonary comorbidity. Table 1 shows that patients in both groups were comparable in terms of distribution of age. Women were much more likely to report a chronic pulmonary comorbidity than men. Patients with a chronic pulmonary comorbidity were somewhat more likely to report hypertension, arthritis, diabetes and kidney disease, though somewhat less likely to report heart failure, though the differences were not statistically significant.

**Table 1:** Summary statistics of CRS patients

	CRS patients without Chronic Pulmonary Comorbidities		CRS patients with Chronic Pulmonary Comorbidities	
	N	%	N	%
Overall	162	100	91	100
Gender				
Female	71	43.8%	55	60.4%
Male	91	56.2%	36	39.6%
Age				
< 40	27	16.7%	13	14.3%
40–49	29	17.9%	19	20.9%
50–59	46	28.4%	25	27.5%
60–69	35	21.6%	25	27.5%
70+	25	15.4%	9	9.9%
Other Chronic Health Conditions (Top 5)				
Hypertension	42	25.9%	26	28.6%
Arthritis	42	25.9%	28	30.8%
Diabetes	20	12.4%	12	13.2%
Heart failure	14	8.6%	6	6.6%
Kidney or renal disease	8	4.9%	6	6.6%

As reported in Table 2, there were a number of significant differences between patients with and without chronic pulmonary comorbidities. Based on the bivariate results, patients with a chronic pulmonary comorbidity had significantly higher SNOT-22, PHQ-9 and PEG-3 scores, than those without a chronic pulmonary comorbidity. The difference in mean SNOT-22 scores between the two groups was greater than the minimal clinically important difference of nine points.

**Table 2:** Average scores of the SNOT-22, PHQ-9, and PEG-3, comparing patients with chronic pulmonary comorbidities to those without

	Without Chronic Pulmonary Comorbidities	With Chronic Pulmonary Comorbidities	*P*-Value
Instrument	Mean (SD)	Mean (SD)	T-Test
SNOT-22	37.7 (21.6)	48.6 (21.9)	< 0.001
Depression (PHQ-9)	4.3 (4.7)	6.2 (6.0)	0.010
Pain (PEG-3)	2.5 (2.6)	3.4 (2.9)	0.009

When age, gender, and the presence of other comorbidities were controlled for in the multivariate linear models, only the differences in the SNOT-22 scores remained significant between those with a chronic pulmonary comorbidity and those without. As detailed in Table 3, age and the presence of other comorbidities eliminated the effect of having a chronic pulmonary comorbidity on the differences between either the PHQ-9 or PEG-3 scores. Although, the results for the PHQ-9 scores were on the threshold of statistical significance (*p* = 0.06).

**Table 3:** Multivariate regression results of the SNOT-22, PEG-3 and PHQ-9 scores among CRS patients waiting for ESS, evaluating the impact of at least one chronic pulmonary comorbidity

	SNOT-22 Score			PEG-3			PHQ-9		
Model Effect	Estimate	SE	*P*-Value	Estimate	SE	*P*-Value	Estimate	SE	*P*-Value
Intercept	18.3	4.5	< 0.001	0.53	0.15	< 0.001	1.03	0.17	< 0.001
Gender									
Female	4.7	2.6	0.070	0.16	0.09	0.056	−0.07	0.10	0.472
Male (Ref.)									
Age									
< 40	13.9	5.0	0.005	0.49	0.17	0.003	0.46	0.19	0.015
40–49	19.9	4.7	< 0.001	0.47	0.16	0.003	0.52	0.18	0.003
50–59	13.5	4.4	0.002	0.48	0.15	0.001	0.39	0.17	0.020
60–69	12.9	4.4	0.003	0.21	0.15	0.150	0.16	0.17	0.358
70+ (Ref.)									
Chronic Pulmonary Comorbidity									
Yes	8.1	2.7	0.002	0.14	0.09	0.127	0.20	0.11	0.065
No (ref.)									
Other Comorbidity Count									
0 (Ref.)									
1	2.0	3.3	0.547	0.29	0.11	0.007	0.32	0.13	0.013
2 or more	13.6	3.2	< 0.001	0.64	0.11	< 0.001	0.76	0.12	< 0.001

## Discussion

This study set out to examine the association between chronic pulmonary conditions and the quality of life in CRS patients. We observed that patients with asthma, emphysema or chronic bronchitis reported significantly more severe CRS-specific symptoms compared to those who did not report having these conditions.

The concomitant relationship between lower airway disease and upper airway symptoms suggests a mechanism of inflammation that targets all respiratory mucosa and elevates the patient’s overall inflammatory load. Previous studies examining this relationship report comparable results [15, 16]. Alobid et al. looked at the specific relationship between in asthma, aspirin sensitivity and CRS patients with nasal polyposis and found that asthmatic patients with nasal polyps had worse quality of life than non-asthmatic patients [17]. Other authors have found the severity of CRS is correlated with the presence of Asthma [18]. More recent studies have investigated the relationship between asthma and CRS in further detail. Multiple authors have found that severity of asthma, as measured by a validated score or number of exacerbations, is correlated with severity of CRS symptoms. This suggests that the clinical status of asthma, not merely the presence of asthma, impacts CRS severity [19, 20].

Additionally, data from the respiratory literature examines this ‘unified airway phenomenon’ from a lower airway perspective, showing that patients with moderate to severe COPD also have higher SNOT 20 scores, compared with those with mild COPD [21]. When assessing overall quality-of-life metrics, the literature suggests that both upper and lower airway symptoms substantially impact quality of life [22, 23]. The results reported in this study add to this knowledge, encompassing CRS patients with and without nasal polyposis and a larger class of lower airway diseases. What is still unclear is whether the association between the upper and lower airway disease is an additive or synergistic effect.

The results from this study also suggest a complex relationship between upper and lower respiratory disease and depression. While the results were not statistically significant, this study’s use of a sensitive instrument for depression found that depression scores were higher among patients with a chronic pulmonary comorbidity. This finding is supported by other studies that examine the relationship between chronic conditions and depression. It has been observed that the prevalence of depression is higher among patients with COPD and asthma [24, 25]. Anxiety has also been shown to occur in higher prevalence in CRS patients and results in worse quality of life, as well as reduced improvement following ESS [26].

Since the rate of depression among people with CRS reportedly exceeds 30% [27], the findings of this study are potentially relevant given that, in the VCH region, wait times for elective ESS can exceed 6 months. The results from this study should draw attention to potential gaps in mental health interventions for patients with CRS awaiting surgery. Targeted interventions could be used to improve the mental health of these patients. These opportunities are particularly salient for CRS patients with a chronic pulmonary comorbidity where this study showed a much higher symptom burden, but only modestly higher depression scores.

There are a number of limitations of this study. The presence of asthma, emphysema, or chronic bronchitis was patient-reported, and not clinically verified. We believe that this reflects the predominant way in which this data is obtained during a clinical encounter, and thus it is a reasonable surrogate marker. If the evidence is based on data that can be simply obtained on history, this information can be used for practical point of care management, such as increased symptom management, referral and surgical triage. Patients with Aspirin Exacerbated Respiratory Disease (AERD) were not excluded from the chronic comorbidity group. There is evidence that this unique group of patients has worse CRS related symptomatology and their inclusion may increase the differences observed between our two groups. Finally, it is possible that the findings’ generalizability was undermined by the response rate among potential study participants; and although we did not detect noticeable differences between respondents and non-respondents, it was possible that there were some unmeasured sources of bias attributable to response rate.

The respiratory diseases were pooled, precluding sub-group analysis. Future research could provide insight into whether, individually, these conditions were associated with differential CRS symptom burden. In addition, the next study could add disease severity measures, such as pulmonary function tests, to isolate sources of variability among sub-groups’ levels of self-reported health.

## Conclusion

This study found that among CRS patients waiting for ESS, chronic pulmonary comorbidities are strongly associated with significantly higher symptom burden. Patients with asthma, emphysema, or chronic bronchitis reported significantly higher SNOT-22 scores than compared to those who did not report having these conditions. The difference in SNOT-22 scores was over nine, indicative of clinical significance.

## Abbreviations

COPD: Chronic obstructive pulmonary disease
CRS: Chronic rhinosinusitis
ESS: Endoscopic sinus surgery
PEG-3: Pain outcome test
PHQ-9: Patient Health Questionnaire-9
SNOT-22: Sino-Nasal Outcome Test-22
